# Safety and Efficacy of Baricitinib, Upadacitinib, and Sarilumab Implementation in Moderately-to-Severely Active Rheumatoid Arthritis: A Systematic Review and Meta-Analysis of Current Evidence

**DOI:** 10.7759/cureus.76466

**Published:** 2024-12-27

**Authors:** Giannis Meziridis, Penelope Sidiropoulou, Chrysa Pourzitaki, Anna-Bettina Haidich, Georgia Tsaousi

**Affiliations:** 1 Department of Trauma and Orthopaedics, General Hospital Wolfhagen, Wolfhagen, DEU; 2 Department of Pharmacy, School of Health Sciences, National and Kapodistrian University of Athens, Athens, GRC; 3 Department of Clinical Pharmacology, School of Medicine, Faculty of Health Sciences, Aristotle University of Thessaloniki, Thessaloniki, GRC; 4 Department of Hygiene, Social-Preventive Medicine and Medical Statistics, School of Medicine, Faculty of Health Sciences, Aristotle University of Thessaloniki, Thessaloniki, GRC; 5 Department of Anesthesiology and Intensive Care, School of Medicine, Faculty of Health Sciences, Aristotle University of Thessaloniki, Thessaloniki, GRC

**Keywords:** baricitinib, interleukin-6 inhibitors, jak inhibitors, meta-analysis, rheumatoid arthritis, sarilumab, systematic review, upadacitinib

## Abstract

This study aims to assess the efficacy and safety of newly approved Janus kinase (JAK) and interleukin-6 (IL-6) inhibitors in patients with moderately-to-severely active rheumatoid arthritis (RA) with inadequate response to or intolerance of conventional disease-modifying antirheumatic drugs. We conducted a systematic review and meta-analysis of all placebo-controlled randomized trials assessing baricitinib, sarilumab, and upadacitinib treatment in RA, published in PubMed and CENTRAL (Cochrane Central Register of Controlled Trials) databases up to October 2023. The study outcomes involved the American College of Rheumatology (ACR) 20%, 50%, and 70% responses, Health Assessment Questionnaire-Disability Index (HAQ-DI), Disease Activity Score in 28 joints (DAS28), serious adverse events, and adverse events leading to drug discontinuation. Twelve randomized controlled trials enrolling 5875 patients were selected for final analysis. Pooled analysis revealed that the implementation of baricitinib (RR = 1.77; 95% CI = 1.58-1.97; I^2 ^= 21%), sarilumab (RR = 1.60; 95% CI = 1.33-1.93; I^2 ^= 0%), and upadacitinib (RR = 1.99; 95% CI = 1.81-2.20; I^2^ = 15%) was associated with notable therapeutic improvement in RA patients as determined by the ACR20. Considering other efficacy outcomes (ACR50, ACR70, DAS28, HAQ-D), all tested interventions demonstrated a superiority to placebo. None of the tested treatment modalities incurred a higher risk of serious adverse events compared to placebo, yet drug discontinuation was more commonly encountered in baricitinib-treated patients. The newly approved JAK and IL-6 inhibitors, namely, baricitinib, upadacitinib, and sarilumab, seem to be effective in alleviating RA-induced clinical implications and thus improving quality of life. Nonetheless, safety issues could be a matter of concern in baricitinib use, while upadacitinib and sarilumab present an acceptable safety profile.

## Introduction and background

Rheumatoid arthritis (RA) is a chronic, progressive inflammatory disease defined as a symmetric polyarthritis of joints that may lead to joint and periarticular structural damage. Over and above, underlying systemic inflammation is further implicated by distal organic systems compromise [1]. Typically, it is characterized by pain and morning stiffness in multiple joints lasting over an hour, which progresses to considerable morbidity and mortality if inadequately treated [2]. The early, aggressive treatment of RA seems to lead to superior clinical responses and radiographic benefits [3]. Over the past two decades, the improved understanding of the involved pathophysiology, the implementation of certain tools for reliable disease assessment, the appropriate use of established treatment modalities, as well as the development of new drugs have contributed to the considerable improvement of life quality of the affected patients [4].

Conventional synthetic disease-modifying antirheumatic drugs (csDMARDs), such as methotrexate, are the cornerstone of RA treatment due to their effectiveness and tolerability, joined with their potential to improve the efficacy of biologic and non-biologic medications. Nonetheless, it is estimated that only half of the patients on methotrexate alone will see a sufficient decline in disease severity or symptom remission [4].

More recently, several biologic and non-biologic agents targeting inflammatory mediators in RA have been widely used. Targeted synthetic disease-modifying antirheumatic drugs (tsDMARDs) were created mainly to target the Janus kinase/signal transducer and activator of transcription (JAK-STAT) pathway, a crucial stage in the cytokine-mediated production of inflammatory responses [5]. Janus kinase (JAK) inhibition prevents the signal transduction that interferons, granulocyte-macrophage colony-stimulating factor (GM-CSF), interleukins, common gamma chain cytokines, leptin, or erythropoietin use to activate cells [6].

Interleukin 6 (IL-6) is a major contributor to the development of RA, mainly acting through systematic inflammatory and bone-destructive cascade promotion. Approved IL-6 receptor (IL-6R) inhibitors tocilizumab and sarilumab have shown good effectiveness and tolerance as monotherapy or combination treatment in RA patients who do not respond well to csDMARDs [4,7].

Current guidelines suggest adding a biologic disease-modifying antirheumatic drug (bDMARD) as a primary therapeutic option in cases where the initial csDMARD therapy fails to provide an adequate response, while tsDMARDs may also be implemented in this phase after the risks of major adverse cardiovascular effects (MACEs), malignancies, and/or thromboembolic events have been carefully considered. Over and above, monotherapy with an IL-6 pathway inhibitor or a tsDMARD is advised, which may have some advantages over other bDMARDs, if the patient is not suited for csDMARD co-therapy [7].

As a result, clinicians require additional data to determine which medication is appropriate for each individual. It should be underlined that the differences in the reported efficacy and safety concerns between the newly approved second-line therapeutic agents are unclear while more evidence is needed to make the most appropriate selection [8]. Two recently published studies attempted a thorough evaluation and data synthesis of current evidence relevant to implementing JAK inhibitors or bDMARDs in RA cases to determine their pooled efficacy and safety to placebo, yet the IL-6R inhibitor sarilumab was not incorporated in these analyses [9]. Considering that JAK inhibitors baricitinib and upadacitinib, as well as the IL-6R inhibitor sarilumab, have been recently approved by the FDA for RA treatment, an insight into these novel therapeutic agents seems to be in order.

Thus this systematic review and meta-analysis was conducted to shed more light on the efficacy and safety profile of baricitinib, upadacitinib, and sarilumab - as head-to-head comparisons - in patients with moderately-to-severely active RA demonstrating inadequate response to or intolerance of csDMARDs, and is based in part on a Master of Science (MSc) thesis research conducted by the authors of the current article [10]. The efficacy was tested using the standardized criteria for improvement in symptoms and RA treatment response (ACR20, ACR50, and ACR70, as defined by the American College of Rheumatology) as primary outcomes, while the disease activity measures (Disease Activity Score in 28 joints, DAS28), health-related quality of life (Health Assessment Questionnaire-Disability Index, HAQ-DI), as well as adverse events (serious adverse events, adverse events leading to drug discontinuation) served as secondary outcomes.

## Review

Methods

Search Strategy

This systematic review and meta-analysis was conducted in compliance with the Preferred Reporting Items for Systematic Reviews and Meta-Analyses of Individual Participant Data (PRISMA-IPD) guidelines [11]. The study protocol was publicly registered in the International Prospective Register of Systematic Reviews (PROSPERO) database (CRD42022353041).

We performed a systematic search in PubMed and CENTRAL (Cochrane Central Register of Controlled Trials) databases, as well as in clinical registries until the 20th of August 2024, to detect all randomized control trials (RCTs) pertinent to the administration of JAK inhibitors, namely, baricitinib and upadacitinib, as well as the IL-6R inhibitor sarilumab in patients suffering from RA. For literature search purposes, medical subject heading (MesH) terms, including “rheumatoid arthritis”, “monoclonal antibodies”, “JAK inhibitor”, “anti-interleukin 6”, “upadacitinib”, “ABT-494”, “rinvoq”, “baricitinib”, “LY3009104”, “INCB028050”, “sarilumab”, “REGN88”, and “Kevzara”, with “AND” and “OR” as Boolean terms, were applied into the databases to retrieve articles relevant to the objectives of this review.

Studies Selection

The retrieved articles meeting the following criteria were considered eligible for inclusion in this review: (1) phase III, placebo-controlled, RCTs with a follow-up period longer than 12 weeks; (2) adult patients (age >18 years) who had been diagnosed according to the criteria illustrated by the American College of Rheumatology (ACR) meeting or the ACR/European League Against Rheumatism (EULAR) criteria [12]; (3) administration of JAK inhibitors (baricitinib, upadacitinib) or IL-6 inhibitors (sarilumab), either alone or in combination with any disease-modifying antirheumatic drugs (DMARDs) versus placebo, in the approved dose and formulation; (4) complete data about outcomes including the ACR 20% (ACR20) and adverse events; (5) full-text publications in the English language.

After duplicate studies removal, two investigators (G.M. and P.S.) independently screened and assessed titles and abstracts of the retrieved articles to identify those being potential candidates for inclusion in this review, while the full-text assessment was reserved should clinical relevance not be ascertained from the title or abstract. Any potential disagreement over eligibility was resolved by consensus among the investigators. In a further attempt to avoid missing any relevant publications, the reference lists from the selected papers were searched in detail.

Data Extraction and Outcome Measures

The primary outcome measure was the clinical improvement of RA following the use of JAK and IL-6R inhibitors as assessed by standardized criteria, namely, ACR20 at 12 and 24 weeks, and ACR50 or ACR70 at 12 weeks. Secondary study outcomes investigated the impact of the implemented practice on adverse events, disease activity measures (DAS28), and health-related quality of life (HAQ-DI).

Using spreadsheet software, a pre-piloted abstraction form specifically developed for this review was created to record all relevant details. Two independent reviewers collected the data of interest. The abstracted data encompassed publication details (author, year of publication, Clinical Trials registration number, number of participants, age, sex, disease duration, RA‐related baseline data), study arms (type of intervention and dosage), concomitant medications, length of follow-up time, and findings relevant to the primary or secondary outcomes. We attempted to contact the original investigators if any information required for the analysis was lacking.

Risk of Bias Assessment

In an attempt to critically appraise the quality of the incorporated RCTs, the Cochrane Collaboration Risk of Bias Tool 2 (RoB2) was implemented to assess the risk of bias (sequence generation, allocation sequence concealment, blinding of participants and personnel, blinding of outcome assessment, incomplete outcome data, selective outcome reporting, and other potential threats to validity). Each item was classified as a low, some concerns, or a high risk of bias. To minimize the impact of subjective interpretation, the methodological quality assigned to each trial was adjudicated by two reviewers (G.M. and G.T.) independently. Any discrepancies encountered after evidence ratings were resolved by consensus.

Synthesis of Results and Statistical Analysis

Data extracted from each study were pooled and included in the meta-analysis. The criterion for inclusion in the study was the retrieval of at least two RCTs for each intervention. Data were analyzed using an intention‐to‐treat model, in case of data availability. Continuous data were analyzed as a mean difference (MD) with 95% confidence intervals. Dichotomous data were reported as a Mantel-Haenszel risk ratio (RR) with 95% confidence intervals. A random-effects model was selected as an appropriate summary measure for analyses. Heterogeneity was quantified using I^2^ (> 50% indicates evidence of notable heterogeneity). Comparison-adjusted funnel plots were used to detect potential publication bias if there were at least 10 studies included in the analysis. The Review Manager 5.3 software (Cochrane Collaboration, London, UK) was used to perform the meta-analysis.

The GRADE (Grading of Recommendations Assessment, Development, and Evaluation) approach of Cochrane Collaboration was used to assess the overall certainty in pooled diagnostic impact estimations. The overall confidence level in effect estimates was classified as high, moderate, low, or extremely low. The guideline-building tool (gradepro.org) generated a GRADE evidence profile for each parameter.

Results

Study Characteristics

**Figure 1:**
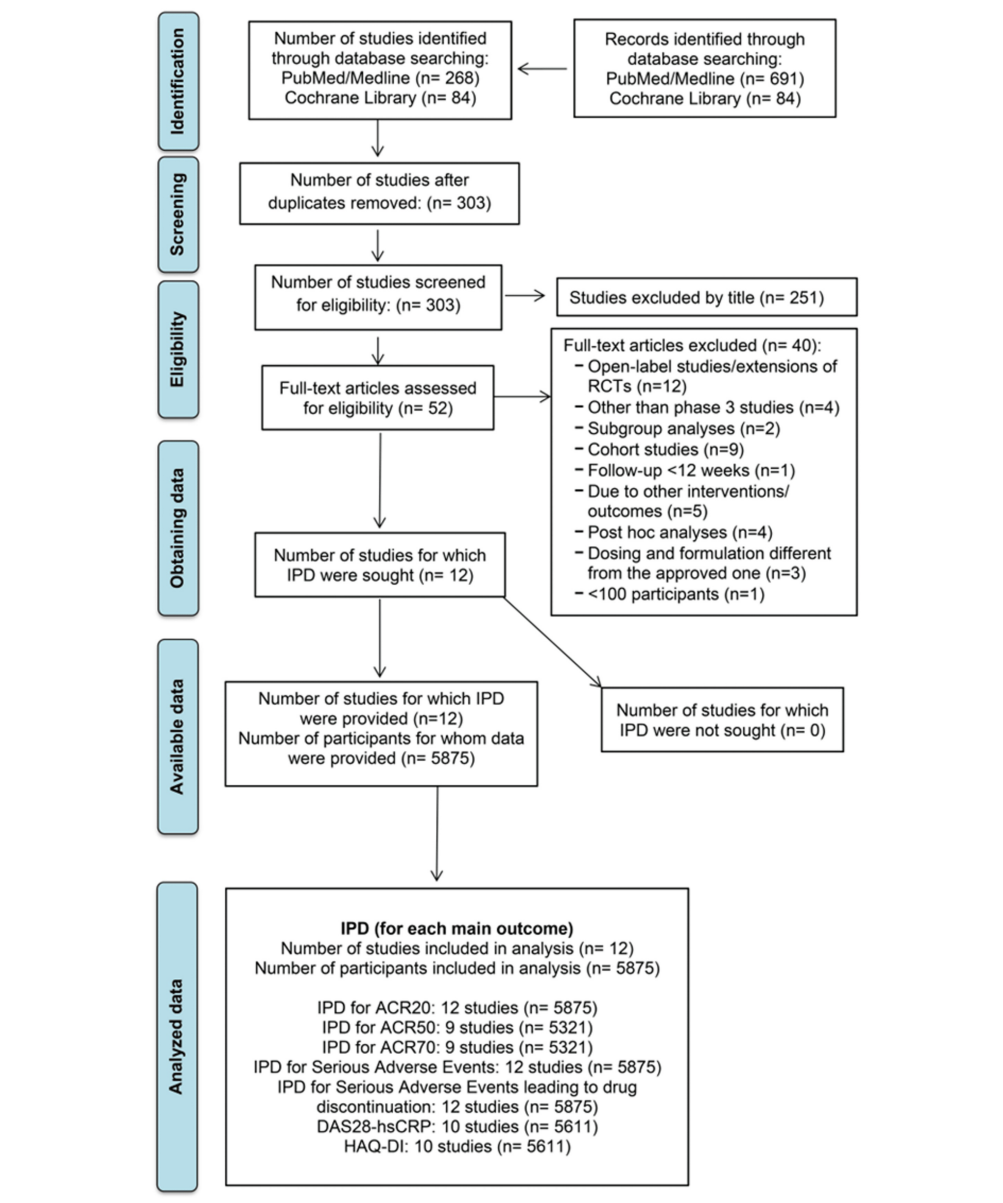
Flow diagram showing the results of the search and reasons for the exclusion of studies according to PRISMA-IPD guidelines. PRISMA-IPD: Preferred Reporting Items for Systematic Reviews and Meta-Analyses of Individual Participant Data; RCTs: randomized control trials; IPD: individual participant data; ACR: American College of Rheumatology; DAS28: Disease Activity Score in 28 joints; hsCRP: high-sensitivity C-reactive protein; HAQ-DI: Health Assessment Questionnaire-Disability Index.

We identified 775 records related to moderately-to-severely active RA as assessed by DAS28, inadequate response or intolerance to csDMARDs, and the implementation of baricitinib, upadacitinib, or sarilumab in the treatment regimen. After the “clinical trials” filter was applied, 352 records resulted. Following de‐duplication, we screened 303 records by title and abstract, from which 251 papers were excluded. From the 52 records that remained for full-text articles assessment, 40 records were excluded. Finally, 12 RCTs [13-24] enrolling a total of 5875 participants fulfilled the eligibility criteria and were included in the qualitative and quantitative analysis. The literature review process is summarized in Figure 1, while the detailed characteristics of the selected studies are reported in Table 1.

**Table 1:** Primary and secondary outcomes findings of the included studies. RA: rheumatoid Arthritis; MTX: methotrexate; ACR: American College of Rheumatology; DAS28: Disease Activity Score in 28 joints; hsCRP: high-sensitivity C-reactive protein; CRP: C-reactive protein; HAQ-DI: Health Assessment Questionnaire-Disability Index; sc: subcutaneous; q: each; wk: week; NR: not reported; PBO: placebo; vs: versus; pts: patients; cDMARD: conventional disease-modifying antirheumatic drugs. * Expressed as number (%) of patients; median (range), and mean (SD). ** Expressed as mean (SD) or mean (95% CI).

Study ID	No. of patients	Interventions	Primary outcomes			Secondary outcomes				
			ACR20*	ACR50*	ACR70*	Serious adverse events*	Infections*	Drug discontinuation due to adverse effects*	DAS28-hsCRP**	HAQ-DI**
Burmester et al. (2018) [13]	455	PBO vs upadacitinib 15 mg/d	Week 12: 35.7% vs 63.8%	Week 12: 14.9% vs 38.0%	Week 12: 5.9% vs 20.8%	2.3% vs 4.1%	Serious: 0.45% vs 0.45%	2.7% vs 0.9%	Week 12: -1.02 (-1.22 to -0.82) vs -2.2 (-2.40 to -2.00)	Week 12: -0.25 (-0.34 to -0.17) vs -0.59 (-0.67 to -0.51)
							Upper respiratory tract: 4.1% vs 5.5%		DAS28-CRP <2.6 (wk 12): 4% vs 1.4%	
Dougados et al. (2017) [14]	159	PBO + cDMARD vs baricitinib 4 mg/d + cDMARD	Week 12: 39.5% vs 61.7%	Week 12: 12.7% vs 33.5%	Week 12: 3.1% vs 18.1%	5.7% vs 5.3%	Serious: 2.2% vs 2.6%	3.07% vs 5.3%	Week 12: -1.05 (1.23) vs -1.91 (1.21)	Week 12: -0.3 (0.45) vs -0.52 (0.59)
				Week 24: 21.5% vs 44.1%	Week 24: 7.9% vs 24.2%		Upper respiratory tract: 7.9% vs 0.5%. Bronchitis: 4.8% vs 3.1%		DAS28-CRP <2.6 (wk 24): 1.1% vs 3.1%	
Fleischmann et al. (2019) [15]	227	PBO vs upadacitinib 15 mg/d	Week 12: 36.4% vs 70.5%	Week 12: 14.9% vs 45.2%	Week 12: 4.9% vs 24.9%	2.9% vs 3.7%	Serious: 1.1% vs 2.3%	2.6% vs 3.4%	Week 12: -1.15 (-1.27 to -1.02) vs -2.48 (-2.61 to -2.34)	Week 12: -0.28 (-0.34 to -0.23) vs -0.6 (-0.65 to -0.54)
							Nasopharyngitis: 2.9% vs 5.5%		DAS28-CRP <2.6 (wk 12): 6% vs 2.9%	
							Upper respiratory tract: 3.7% vs 5.7%			
Fleischmann et al. (2017) [16]	584	Baricitinib 4 mg/d + MTX 10-20 mg/wk vs baricitinib 4 mg/d + PBO vs MTX 10-20 mg/wk	Week 24: 78.1% vs 76.7% vs 61.9%	Week 24: 63.3% vs 59.7% vs 43.3%	Week 24: 39.5% vs 42.1% vs 21.4%	7.9% vs 7.5% vs 9.5%	Serious: 2.3% vs 3.8% vs 4.3%	11.1% vs 6.3% vs 3.8%	Week 24: -2.82 (1.6) vs -2.74 (1.4) vs -2.01 (1.5)	Week 24: -0.92 (0.74) vs -1.01 (0.74) vs 0.73 (0.71)
			Week 52: 72.6% vs 73% vs 55.7%	Week 52: 61.9% vs 57.2% vs 37.6%	Week 52: 46% vs 42.1% vs 25.2%		Upper respiratory tract: 7.4% vs 7.5% vs 7.1%. Bronchitis: 4.2% vs 3.1% vs 1.9%		DAS28-hsCRP <2.6 (wk 24): 0.4% vs 40.2% vs 23.8%	
Fleischmann et al. (2017) [17]	975	PBO + cDMARD vs sarilumab 200 mg q2wk + cDMARD	Week 12: 37.6% vs 62.5%	Week 12: 13.3% vs 33.2%	Week 12: 2.2% vs 14.7%	3.3% vs 5.4%	Serious: 1.6% vs 1.1%	4.97% vs 9.2%	Week 12: -0.97 (0.10) vs -2.45 (0.10)	Week 12: -0.26 (0.04) vs -0.47 (0.04)
			Week 24: 33.7% vs 60.9%	Week 24: 18.2% vs 40.8%	Week 24: 7.2% vs 16.3%		Nasopharyngitis: 4.9% vs 3.8%		Week 24: -1.38 (0.12) vs -2.82 (0.11)	Week 24: -0.34 (0.05) vs -0.58 (0.05)
							Urinary tract: 6.6% vs 7.07%			
Genovese et al. (2016) [18]	210	PBO + cDMARD vs baricitinib 4 mg/d + cDMARD	Week 12: 27.3% vs 55.4%	Week 12: 28.2% vs 8.0%	Week 12: 2.3% vs 11.3%	7.9% vs 10.7%	Serious: 2.8% vs 3.9%	3.9% vs 5.6%	Week 12: -0.85 (1.2) vs -1.81 (1.4)	Week 12: -0.2 (0.5) vs -0.42 (0.5)
			Week 24: 27.3% vs 46.3%	Week 24: 13.1% vs 29.4%	Week 24: 3.4% vs 16.9%		Upper respiratory tract: 4.5% vs 5.1%. Bronchitis: 3.4% vs 5.6%		DAS28-CRP <2.6 (wk 24): 5.7% vs 10.7%	
							Nasopharyngitis: 3.9% vs 5.1%			
Huizinga et al. (2014) [19]	487	PBO + MTX vs sarilumab 200 mg (sc) q2wk + MTX	Week 12: 46.2% vs 65.4%	NR	NR	3.9% vs 0	Serious: 0	7.7% vs 1.9%	NR	NR
Tanaka et al. (2019) [20]	488	PBO + MTX vs sarilumab 200 mg (sc) q2wk + MTX	Week 24: 14.8% vs 57.5%	NR	NR	4.4% vs 6.2%	Serious: 1.2% vs 0	6.17% vs 10%	NR	NR
							Nasopharyngitis: 14.8% vs 28.7%			
							Upper respiratory tract: 4.9% vs 8.7%			
Taylor et al. (2017) [21]	215	PBO + MTX vs baricitinib 4 mg/d + MTX	Week 12: 40.2% vs 69.6%	Week 12: 16.8% vs 45.0%	Week 12: 4.7% vs 18.9%	5.3% vs 5.3%	Serious: 1.4% vs 1.2%	5.1% vs 6.9%	Week 12: -1.01 (1.12) vs -2.27 1.22)	Week 12: -0.33 (0.51) vs -0.65 (0.59)
				Week 24: 19.3% vs 50.5%	Week 24: 8.0% vs 29.8%		Upper respiratory tract: 2.9% vs 3.1%		DAS28-CRP <2.6 (wk 24): 7.8% vs 34.5%	
							Urinary tract: 3.3% vs 4.3%			
Yang et al. (2020) [22]		PBO + MTX vs baricitinib 4 mg/d + MTX	Week 12: 28.3% vs 58.6%	NR	NR	2.7% vs 2.7%	Serious: 0.7% vs 1.4%	2.07% vs 1.4%	Week 12: -0.94 (1.04) vs -1.89 (1.14)	Week 12: -0.35 (0.5) vs -0.57 (0.5)
							Upper respiratory tract: 15.2% vs 19.3%			
							Nasopharyngitis: 4.14% vs 6.9%			
Zeng et al. (2021) [23]	527	PBO vs upadacitinib 15 mg/d	Week 12: 31.4% vs 71.6%	Week 12: 8.3% vs 40.8%	Week 12: 3.6% vs 21.3%	2.9 vs 7.1%	Serious: 0.6% vs 2.4%	2.4% vs 4.1%	Week 12: -0.95 (-1.16 to -0.74) vs -2.56 (-2.76 to -2.36)	Week 12: -0.18 (-0.28 to -0.09) vs -0.62 (-0.71 to -0.54)
							Upper respiratory tract: 6.5% vs 9.4%		DAS28-CRP <2.6 (wk 12): 5.3% vs 46.1%	
Genovese et al. (2018) [24]	228	PBO vs upadacitinib 15 mg/d	Week 12: 28.4% vs 64.6%	Week 12: 11.8% vs 34.1%	Week 12: 6.5% vs 11.6%	0 vs 4.9%	Serious: 0 vs 0.6%	4.14% vs 1.8%	Week 12: -1.02 (-1.23 to -0.8) vs -2.31 (-2.52 to -2.10)	Week 12: -0.17 (-0.26 to -0.08) vs -0.39 (-0.48 to -0.30)
							Upper respiratory tract: 7.7% vs 16.1%			

Quality of Studies

**Figure 2:**
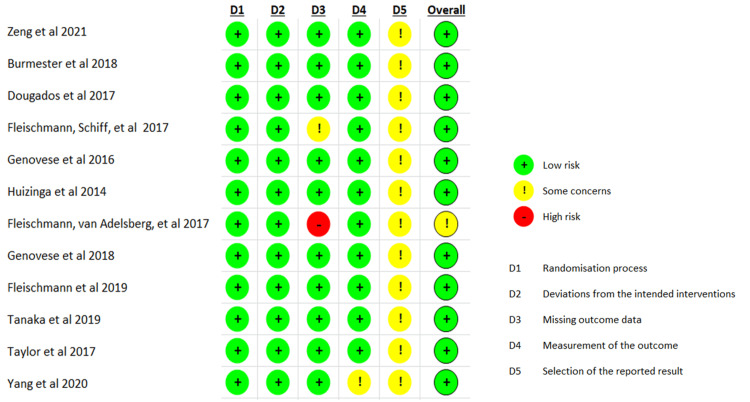
Risk of bias assessment with RoB-2 tool. RoB-2: Risk of Bias 2. References [13-24].

The majority of the included studies were of moderate to low risk of bias (Figure 2). In detail, the randomization method was clearly described by all RCTs, and thus the risk of bias was graded as low. Considering that the sequence generation was performed by an interactive voice response system and the allocation was concealed from the sponsor, investigators, and patients until data analysis was performed, all included studies were considered at low risk of bias for allocation concealment. Although some studies did not describe the allocation concealment process explicitly, they were characterized as quadruple masking (participant, care provider, investigator, outcomes assessor), and thus they were also characterized as low risk of bias. All RCTs included adequate descriptions of blinding and were assessed as low risk of performance bias. Regarding the detection bias, merely one study [22] failed to identify the blinding process. All of the included studies, except two [16,17], described methods to deal with withdrawals and loss to follow‐up and were considered at low risk of attrition bias. Regarding the reporting bias, it was considered to be low on the basis that a wide range of outcomes were reported in most studies making the probability of selective reporting unlikely. About other biases that had the potential to affect the results, the risk was evaluated as ‘’some concerns’’ because a pharmaceutical company sponsored all of the included studies, so they are more likely to favor the interventions. Publication bias analysis was not pursued because the number of included studies was inadequate to assess a funnel plot properly.

Efficacy of the Interventions

ACR response: In general, all interventions showed effectiveness in lowering disease activity. In detail, all RCTs, except two [16,20], reported an improvement of ACR20 at 12 weeks. ACR20 response rates were considerably higher following therapy (RR = 1.85; 95% CI = 1.70-2.00; I^2^ = 35%). ACR20 response at week 12 was higher for upadacitinib than other interventions, as patients were 1.99 times more likely to improve at least 20% compared to the patients in the placebo group (RR = 1.99; 95% CI = 1.81-2.20; I^2 ^= 15%). Sarilumab seemed to be the least effective drug in terms of ACR20 at 12 weeks (RR = 1.60; 95% CI = 1.33-1.93; I^2 ^= 0%). At 24 weeks, ACR20 improvement, which was referred for baricitinib and sarilumab studies, was superior for patients in the sarilumab group compared to placebo (RR = 2.56; 95% CI = 1.22-5.38; I^2^ = 84%). Both baricitinib and upadacitinib were more effective than placebo on ACR50 and ACR70 at 12 weeks. The higher response was recorded in the upadacitinib subgroup for ACR50 (RR = 3.06; 95% CI = 2.49-3.77; I^2^ = 29%) as well as in the baricitinib subgroup for ACR70 (RR = 4.46; 95% CI = 3.11-6.39; I^2^ = 0%) at week 12, with high certainty of the evidence for both outcomes (Tables 2-4).

**Table 2:** Summary of rating the certainty of evidence for upadacitinib according to GRADE. * The risk in the intervention group (and its 95% CI) is based on the assumed risk in the comparison group and the relative effect of the intervention (and its 95% CI). GRADE Working Group grades of evidence. High certainty: We are very confident that the true effect lies close to that of the estimate of the effect. Moderate certainty: We are moderately confident in the effect estimate; the true effect is likely to be close to the estimate of the effect, but there is a possibility that it is substantially different. Low certainty: Our confidence in the effect estimate is limited; the true effect may be substantially different from the estimate of the effect. Very low certainty: We have very little confidence in the effect estimate; the true effect is likely to be substantially different from the estimate of the effect. CI: confidence interval; RR: risk ratio; ACR: American College of Rheumatology; RCT: randomized control trial; GRADE: Grading of Recommendations Assessment, Development, and Evaluation.

Upadacitinib compared to placebo for rheumatoid arthritis						
Patient or population: rheumatoid arthritis. Intervention: upadacitinib. Comparison: placebo						
Outcomes	Anticipated absolute effects^*^ (95% CI)		Relative effect (95% CI)	No. of participants (studies)	Certainty of the evidence (GRADE)	Comments
	Risk with placebo	Risk with upadacitinib				
ACR20 follow-up: 12 weeks	344 per 1.000	685 per 1.000 (623 to 758)	RR = 1.99 (1.81 to 2.20)	2415 (4 RCTs)	⨁⨁⨁◯ Moderate	Downgraded one level for study limitations: The studies have an unclear risk of at least one type of bias and publication bias is suspected.
ACR50 follow-up: 12 weeks	136 per 1.000	416 per 1.000 (338 to 511)	RR = 3.06 (2.49 to 3.77)	2418 (4 RCTs)	⨁⨁⨁⨁ High	Downgraded one level for study limitations: The studies have an unclear risk of at least one type of bias and publication bias is suspected. Upgraded one level due to large effect.
ACR70 follow-up: 12 weeks	51 per 1.000	194 per 1.000 (120 to 313)	RR = 3.79 (2.35 to 6.12)	2415 (4 RCTs)	⨁⨁⨁◯ Moderate	Downgraded one level for study limitations: The studies have an unclear risk of at least one type of bias and publication bias is suspected. Upgraded one level due to large effect. Downgraded one level due to inconsistency (high heterogeneity).
Serious adverse events	24 per 1.000	43 per 1.000 (24 to 79)	RR = 1.81 (0.99 to 3.31)	2415 (4 RCTs)	⨁⨁⨁◯ Moderate	Downgraded one level for study limitations: The studies have an unclear risk of at least one type of bias and publication bias is suspected.
Serious adverse events leading to discontinuation	28 per 1.000	26 per 1.000 (13 to 52)	RR = 0.92 (0.46 to 1.84)	2416 (4 RCTs)	⨁⨁◯◯ Low	Downgraded one level for study limitations: The studies have an unclear risk of at least one type of bias and publication bias is suspected. Downgraded one level due to imprecision bias.

**Table 3:** Summary of rating the certainty of evidence for sarilumab according to GRADE. * The risk in the intervention group (and its 95% CI) is based on the assumed risk in the comparison group and the relative effect of the intervention (and its 95% CI). GRADE Working Group grades of evidence. High certainty: We are very confident that the true effect lies close to that of the estimate of the effect. Moderate certainty: We are moderately confident in the effect estimate; the true effect is likely to be close to the estimate of the effect, but there is a possibility that it is substantially different. Low certainty: Our confidence in the effect estimate is limited; the true effect may be substantially different from the estimate of the effect. Very low certainty: We have very little confidence in the effect estimate; the true effect is likely to be substantially different from the estimate of the effect. CI: confidence interval; RR: risk ratio; ACR: American College of Rheumatology; RCT: randomized control trial; GRADE: Grading of Recommendations Assessment, Development, and Evaluation.

Sarilumab compared to placebo for rheumatoid arthritis						
Patient or population: rheumatoid arthritis. Intervention: sarilumab. Comparison: placebo						
Outcomes	Anticipated absolute effects^*^ (95% CI)		Relative effect (95% CI)	No. of participants (studies)	Certainty of the evidence (GRADE)	Comments
	Risk with placebo	Risk with sarilumab				
ACR20 follow-up: 12 weeks	392 per 1.000	632 per 1.000 (522 to 757)	RR = 1.60 (1.33 to 1.93)	467 (2 RCTs)	⨁⨁⨁◯ Moderate	The studies were graded as having a high and unclear risk of bias. Publication bias is suspected.
ACR20 follow-up: 24 weeks	275 per 1.000	704 per 1.000 (335 to 1.000)	RR = 2.56 (1.22 to 5.38)	526 (2 RCTs)	⨁⨁⨁◯ Moderate	The studies were graded as having a high and unclear risk of bias. Publication bias is suspected. Downgraded one level due to inconsistency: high heterogeneity. Upgraded one level due to a large effect.
Serious adverse events	53 per 1.000	95 per 1.000 (50 to 178)	RR = 1.77 (0.94 to 3.33)	526 (3 RCTs)	⨁⨁◯◯ Low	The studies were graded as having a high and unclear risk of bias. Publication bias is suspected. Downgraded one level due to imprecision bias.
Serious adverse events leading to drug discontinuation	57 per 1.000	79 per 1.000 (35 to 178)	RR = 1.38 (0.61 to 3.11)	630 (3 RCTs)	⨁⨁◯◯ Low	The studies were graded as having a high and unclear risk of bias. Publication bias is suspected. Downgraded one level due to imprecision bias.

**Table 4:** Summary of rating the certainty of evidence for baricitinib according to GRADE. * The risk in the intervention group (and its 95% CI) is based on the assumed risk in the comparison group and the relative effect of the intervention (and its 95% CI). GRADE Working Group grades of evidence. High certainty: We are very confident that the true effect lies close to that of the estimate of the effect. Moderate certainty: We are moderately confident in the effect estimate; the true effect is likely to be close to the estimate of the effect, but there is a possibility that it is substantially different. Low certainty: Our confidence in the effect estimate is limited; the true effect may be substantially different from the estimate of the effect. Very low certainty: We have very little confidence in the effect estimate; the true effect is likely to be substantially different from the estimate of the effect. CI: confidence interval; RR: risk ratio; ACR: American College of Rheumatology; RCT: randomized control trial; GRADE: Grading of Recommendations Assessment, Development, and Evaluation.

Baricitinib compared to placebo for rheumatoid arthritis						
Patient or population: rheumatoid arthritis. Intervention: baricitinib. Comparison: placebo						
Outcomes	Anticipated absolute effects^*^ (95% CI)		Relative effect (95% CI)	No. of participants (studies)	Certainty of the evidence (GRADE)	Comments
	Risk with placebo	Risk with baricitinib				
ACR20 follow-up: 12 weeks	362 per 1.000	640 per 1.000 (571 to 712)	RR 1.77 (1.58 to 1.97)	2073 (4 RCTs)	⨁⨁⨁◯ Moderate	Downgraded one level for study limitations: The studies have an unclear risk of at least one type of bias and publication bias is suspected.
ACR50 follow-up: 12 weeks	140 per 1.000	385 per 1.000 (321 to 461)	RR = 2.75 (2.29 to 3.29)	1783 (3 RCTs)	⨁⨁⨁⨁ High	Downgraded one level for study limitations: The studies have an unclear risk of at least one type of bias and publication bias is suspected. Upgraded one level due to large effect.
ACR70 follow-up: 12 weeks	38 per 1.000	170 per 1.000 (119 to 244)	RR = 4.46 (3.11 to 6.39)	1783 (3 RCTs)	⨁⨁⨁◯ Moderate	Upgraded one level due to large effect. Downgraded one level for study limitations: The studies have an unclear risk of at least one type of bias and publication bias is suspected.
ACR20 follow-up: 24 weeks	411 per 1.000	658 per 1.000 (510 to 847)	RR = 1.60 (1.24 to 2.06)	2208 (4 RCTs)	⨁⨁◯◯ Low	Downgraded one level due to inconsistency (high heterogeneity). Downgraded one level for study limitations: The studies have an unclear risk of at least one type of bias and publication bias is suspected.
Serious adverse events	62 per 1.000	62 per 1.000 (46 to 85)	RR = 1.01 (0.74 to 1.37)	2498 (5 RCTs)	⨁⨁◯◯ Low	Downgraded one level due to imprecision bias. Downgraded one level for study limitations: The studies have an unclear risk of at least one type of bias.
Serious adverse events leading to drug discontinuation	40 per 1.000	65 per 1.000 (46 to 91)	RR = 1.61 (1.14 to 2.28)	2498 (5 RCTs)	⨁⨁⨁◯ Moderate	Downgraded one level for study limitations: The studies have an unclear risk of at least one type of bias and publication bias is suspected.

**Figure 3:**
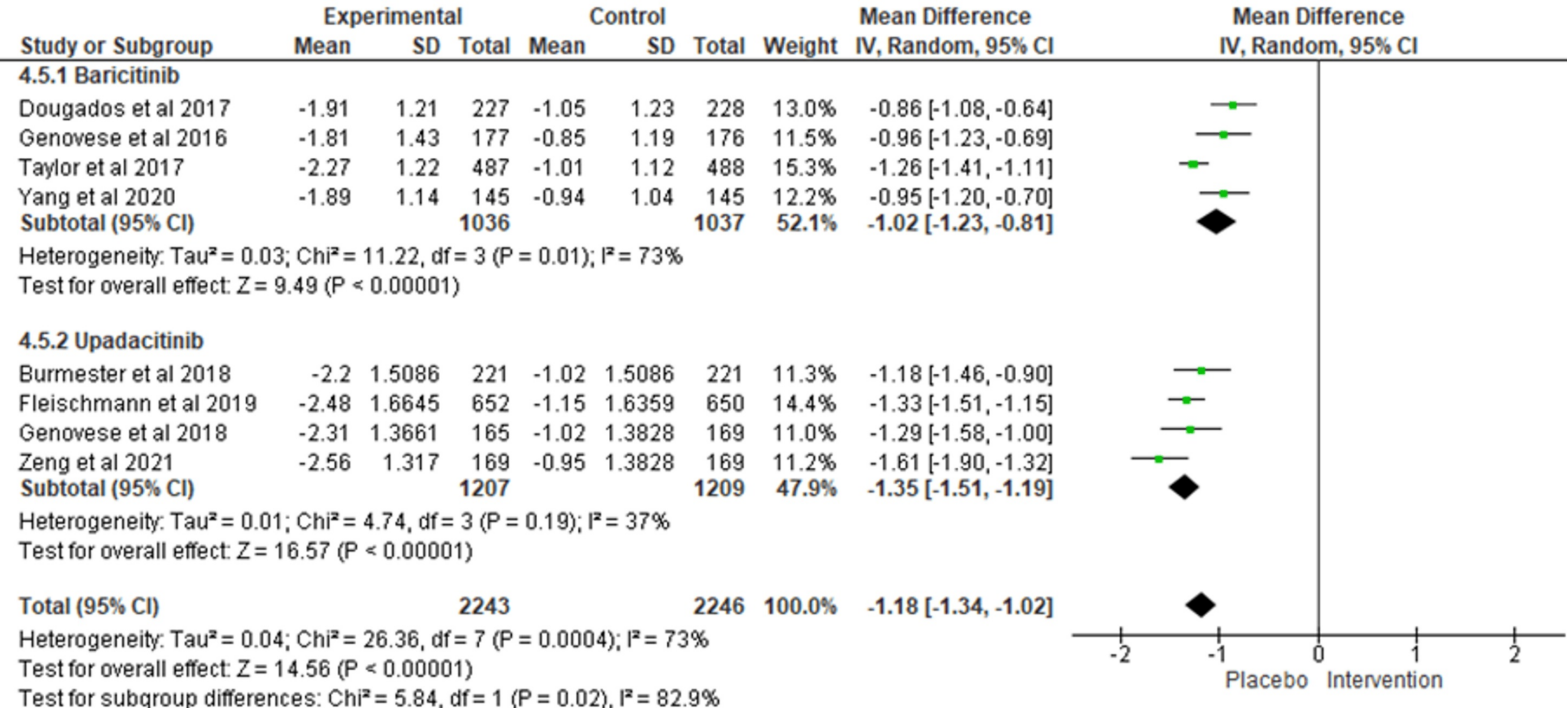
DAS28 at week 12. DAS28: Disease Activity Score in 28 joints.

Disease activity and remission: The administration of both JAK inhibitors (baricitinib and upadacitinib) produced an important decrease in DAS28 in 12 weeks (MD = -1.18; 95% CI = -1.34 to -1.02; I^2 ^= 73%). Improvements in DAS28 score were not mentioned in the results of more than two sarilumab vs. placebo studies, but only one [17] trial. Among the assessed subgroups, upadacitinib was associated with the most significant improvement compared to placebo (MD = -1.35; 95% CI = -1.51 to -1.19; I^2^ = 37%), followed by baricitinib (MD = -1.02; 95% CI = -1.23 to -0.81; I^2^ = 73%) (Figure 3).

**Figure 4:**
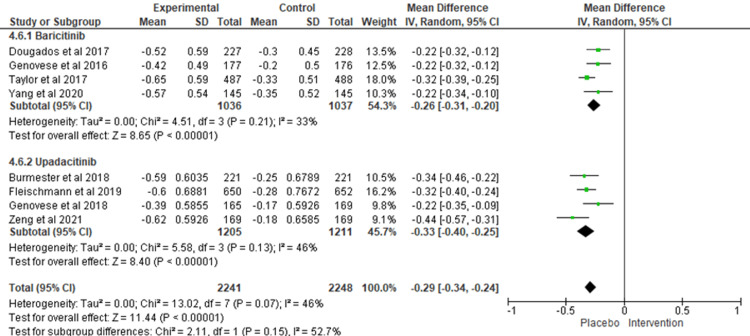
HAD-DI at week 12. HAQ-DI: Health Assessment Questionnaire-Disability Index.

HAQ-DI: The results of the overall pooled analysis for the HAQ-DI score in 12 weeks revealed that both JAK inhibitor treatment groups were linked to significant drops in HAQ-DI values compared to placebo (MD = -0.29; 95% CI = -0.34 to -0.24; I^2^ = 46%). HAQ-DI was not mentioned in the results of more than two sarilumab versus placebo studies, but only in Fleischmann et al.'s trial [17]. Upadacitinib was associated with the most significant improvement in HAQ-DI score (MD = -0.33; 95% CI = ‐0.40 to ‐0.25; I^2^ = 46%) and participants who received upadacitinib rated the change in their disability to be 0.33 points lower on a scale of 0 to 3, compared with those who took placebo. For baricitinib, the change was 0.26 points lower (MD = -0.26; 95% CI = ‐0.31 to ‐0.20; I^2^ =33%) (Figure 4).

Safety of the Interventions

Serious adverse events: The pooled risk of serious adverse events in the intervention groups was comparable to placebo (RR = 1.24; 95% CI = 0.98-1.57; I^2^ = 0%). Upadacitinib (RR = 1.81; 95% CI = 0.99-3.31; I^2^ = 28%) and sarilumab (RR = 1.77; 95% CI = 0.94-3.33; I^2^ = 0%) subgroups demonstrated a tendency to increased risk of serious adverse events, compared to placebo yet this effect was not statistically significant. The baricitinib subgroup showed similar serious adverse events to placebo (RR = 1.01; 95% CI = 0.74-1.37; I^2^ = 0%). The GRADE quality of serious adverse events was judged to be low in baricitinib and sarilumab subgroups due to imprecision bias and study limitations, while in the upadacitinib group, it was graded as moderate (see Tables 2-4).

Adverse events leading to drug discontinuation: Patients treated with baricitinib (RR = 1.61; 95% CI = 1.14-2.28; I^2^ = 0%) were significantly more likely than the other two interventions to discontinue treatment and withdraw from the study due to adverse events compared to patients treated with placebo. On the contrary, in upadacitinib and sarilumab subgroups, no statistically significant association with severe events imposing drug discontinuation compared to placebo could be demonstrated (RR = 1.38; 95% CI = 0.61-3.11; I^2 ^= 33% and RR = 0.92; 95% CI = 0.46-1.84; I^2^ = 37%, respectively). The GRADE quality of this outcome parameter was judged to be low in sarilumab and upadacitinib subgroups, due to imprecision bias and study limitations, while it was graded as moderate in the baricitinib group (see Tables 2-4).

Discussion

This systematic review and meta-analysis investigated the efficacy and safety of the newly approved JAK inhibitors, namely, baricitinib and upadacitinib, as well as the IL-6R inhibitor, sarilumab, in patients with moderately-to-severely active RA and inadequate response to or intolerance of csDMARDs. Our results showed that the two tested JAK inhibitors and the IL-6 inhibitor were more effective compared to placebo in ACR20 at 12 weeks post-treatment commencement. The rest of the efficacy outcomes, namely, ACR50, ACR70, DAS28 score, and HAQ-ID score, were reported only in trials involving JAK inhibitors, in which the aforementioned drugs presented a superior efficacy to placebo. However, safety issues raised a serious concern on the basis that minor and major (leading to drug discontinuation) adverse events were encountered more commonly in the tested treatment modalities.

Considering that the JAK-STAT pathway plays a crucial role in the cytokine-mediated production of inflammatory responses elicited by RA, the JAK inhibitors, namely, baricitinib and upadacitinib, have become a promising pharmacological target for the treatment of RA [5,25-27].

Moreover, bDMARDs, such as IL-6 receptor inhibitors, are implemented as monotherapy or in combination with csDMARDs for the treatment of adults with moderate-to-severe RA, in patients who are intolerant or inadequate responders to csDMARDs [4].

Baricitinib and upadacitinib, as well as the IL-6R inhibitor, sarilumab, constitute novel medications approved by the European Medicine Agency for the treatment of adult patients with moderate to severely active RA with a prior inadequate response or intolerance to methotrexate, with sarilumab demonstrating relatively superior efficacy and safety profile over to JAK inhibitors [28].

Baricitinib, upadacitinib, and sarilumab have all been the subject of several clinical trials aiming to determine their efficacy and safety in treating active RA, yet no head-to-head comparisons of these medications have been conducted [10,29-31].

In our systematic review, data relevant to ACR20 at week 24 were provided only for baricitinib and sarilumab with higher response in sarilumab subgroup but findings should be interpreted with caution due to the small number of studies involved, the moderate certainty of the evidence, and the high risk of bias in one of the studies for sarilumab subgroup and “some concerns” risk of at least one type of bias (sponsorship bias).

Regarding ACR50 and ACR70, these outcomes were reported for baricitinib and upadacitinib subgroups with higher response in the upadacitinib subgroup for ACR50 and baricitinib subgroup for ACR70 and with high certainty of the evidence for both outcomes. Among the baricitinib and upadacitinib groups, the DAS28 score was profoundly improved in the upadacitinib group. Among baricitinib and upadacitinib subgroups, upadacitinib seemed to show the most beneficial effect on HAQ-ID score, yet the improvement following the implementation of both drugs was regarded as clinically significant. The previous results should be interpreted with caution due to the small number of studies involved, especially for sarilumab. Of importance, the certainty of the evidence was evaluated as moderate based on the fact that the studies have “some concerns” risk of at least one type of bias and there is a possibility of publication bias.

Our findings are in line with previous studies conducted in this research area. In detail, current clinical evidence consistently reports the superiority of baricitinib, upadacitinib, and sarilumab in ACR20/50/70, DAS28, and HAQ-DI over placebo. In terms of safety outcomes, an augmented incidence of infections, especially herpes zoster, was reported in the JAK inhibitors group compared to placebo. In particular, Wang et al. [32] suggested that baricitinib and upadacitinib enhanced RA control as determined by ACR20 and HAQ-DI, while adverse events, in particular infections, were more commonly reported in upadacitinib and baricitinib groups compared to placebo. Furthermore, no increased risk of venous thromboembolic events was registered in upadacitinib, whereas relevant data were not available for the use of baricitinib [3]. On the other hand, Kunwar et al. [33] reported that baricitinib was more effective than placebo, and with the exception of the increased incidence of herpes zoster, no notable risk of serious adverse events was identified.

Three recently published studies assessed the efficacy of JAK inhibitors and consistently demonstrated an improved clinical performance, and health-related quality of life compared to placebo [9,10,31]. However, baricitinib and upadacitinib carried a higher risk of infections and adverse events overall, while baricitinib was the only medication linked to a higher risk of herpes zoster. On the other hand, Aly and Furst [34] showed that sarilumab mitigated RA clinical signs, improved functionality, and reduced radiological progression for up to 52 weeks, while the adverse events most commonly encountered were infections and neutropenia. Similarly, Yip and Yim [7], who focused on the role of IL-6R inhibitors in the management of RA, reported that sarilumab demonstrates significant improvements in ACR20 response rates, physical function (measured by the HAQ-DI scores), and remission rates (assessed by the DAS28 and Clinical Disease Activity Index scores) compared to placebo. Furthermore, sarilumab demonstrated a good tolerability and safety profile in clinical trials, with the majority of adverse events being mild to moderate and these results were consistent up to seven years in long-term studies. It also seemed to provide additional benefits in alleviating extra-articular manifestations of RA.

Considering the safety issues in our study, the recorded risk of serious adverse events in all intervention groups was similar to that of the placebo. Although drug discontinuation was imposed more commonly in baricitinib-treated patients, mainly due to the occurrence of MACEs, malignancies, major thromboembolic events, and serious bacterial or viral infections, the risk of serious adverse events occurrence in this treatment modality did not differ from the placebo group. Notably, the administration of upadacitinib and sarilumab was not associated with a higher risk of serious adverse events or drug discontinuation compared to placebo.

The aforementioned findings - in general terms - confirm those of several investigators who failed to identify any particular difference between baricitinib and upadacitinib subgroups to placebo, in terms of serious adverse events and the necessity for drug discontinuations due to adverse effects occurrence [10,30,35]. Notably, Wang et al. [32] recorded an augmented risk of adverse events only in upadacitinib-treated patients, while baricitinib demonstrated a safety profile similar to a placebo. Although Tóth et al. [31] recorded a similar impact of JAK inhibitors on serious adverse effects, they did not clarify the contribution of each treatment regimen in this outcome. It becomes evident that the safety profile of baricitinib and upadacitinib is not fully elucidated yet, and more comprehensive information on this outcome should be provided. Consistent with our study findings, current evidence supports the enhanced tolerance and safety profiles of sarilumab [7]. Long-term usage of this IL-6R inhibitor has resulted in a stable and consistent safety profile, with no serious adverse events to report [7].

Although several earlier meta-analyses examined the safety and effectiveness of JAK or IL-6R inhibitors in the treatment of RA [9,10,30,31,35], we conducted our meta-analysis using more rigorous criteria and incorporating only high-quality evidence as provided by double-blind placebo-controlled RCTs. Over and above, we attempted a direct assessment of three broadly used tsDMARDs and bDMARDs, namely, baricitinib, upadacitinib, and sarilumab.

Nonetheless, several limitations could be acknowledged in our study. Firstly, the number of the included studies was insufficient to establish a firm conclusion regarding the implementation of baricitinib, upadacitinib, and sarilumab, especially for the effect of sarilumab. This was further implicated by the heterogeneity of the available information. Secondly, outcome analysis was not feasible for certain follow-up periods (week 12, week 24, and week 52) due to a lack of consistency of reported data among the incorporated trials. Finally, the findings of this meta-analysis were based on data from RCTs, indicating that they cannot be extrapolated to RA populations commonly encountered in clinical practice, which present a variety of features and treatment regimens.

## Conclusions

This systematic review and meta-analysis indicate that baricitinib, sarilumab, and upadacitinib are equally effective in attenuating clinical signs and symptoms of RA as well as improving health-related quality of life. Nonetheless, safety issues need to be taken into consideration, as an increased necessity of drug discontinuation due to adverse effects was recorded in the baricitinib group compared to placebo, whereas upadacitinib and sarilumab demonstrated an acceptable safety profile. Future research in this area should focus on head-to-head comparisons between active medications to elucidate the most efficacious and tolerable medication for RA and to assess the long-term safety of the available treatment regimens.
